# Editorial commentary on the special issue of cancer research

**DOI:** 10.7555/JBR.39.20250800

**Published:** 2025-09

**Authors:** Editorial Board

Cancer remains one of the leading causes of mortality worldwide, with its complex etiology and heterogeneous progression posing persistent challenges to early diagnosis and effective treatment. In this special issue, we present a collection of studies that delve into the molecular mechanisms, therapeutic resistance, and novel treatment strategies across multiple cancer types, including lung, breast, stomach, and liver cancers. These studies not only advance our understanding of tumor biology but also provide translational insights with potentially significant clinical implications.

Wang *et al*^[1]^ employed large-scale Mendelian randomization to explore the causal relationships between thyroid dysfunction and lung cancer risk. Their findings revealed that both hypothyroidism and hyperthyroidism were causally associated with increased lung cancer risk, with hyperthyroidism also mediating the effect of smoking behavior. This study underscores the importance of endocrine-immune interactions in cancer development and highlights thyroid function as a potentially modifiable risk factor in lung carcinogenesis, particularly among smokers.

In the context of targeted therapy resistance, Lyu *et al*^[2]^ investigated the efficacy of anlotinib in overcoming osimertinib resistance in non-small cell lung cancer. Through both clinical and preclinical models, they demonstrated that the combination of osimertinib and anlotinib effectively reversed osimertinib resistance by inhibiting epithelial-to-mesenchymal transition and angiogenesis. This combination strategy offers a promising approach for managing resistance in patients with EGFR-mutant, non-small cell lung cancer.

Sun *et al*^[3]^ evaluated the timing and dosage of thoracic radiotherapy (TRT) in extensive-stage small cell lung cancer following chemoimmunotherapy. Their retrospective analysis showed that consolidative TRT significantly improved overall and progression-free survival compared with salvage TRT, with low-dose regimens demonstrating comparable efficacy and favorable toxicity profiles. These findings support the integration of early, low-dose TRT into the management of extensive-stage small cell lung cancer.

Huo *et al*^[4]^ elucidated the role of DEC1 in promoting breast cancer bone metastasis *via* transcriptional activation of CXCR4. Their study revealed that DEC1 enhanced the CXCR4/CXCL12 signaling axis within the bone microenvironment, facilitating metastatic colonization. Targeting DEC1 or its downstream effectors may represent a novel strategy for preventing bone metastasis in breast cancer patients.

In gastric cancer research, Han *et al*^[5]^ identified DDR1 as a key oncogenic driver through phosphoproteomic profiling. They demonstrated that *DDR1* overexpression promoted tumor progression *via* inhibition of the Hippo pathway and activation of the YAP-TEAD signaling axis. Importantly, *DDR1*-overexpressing tumors showed increased sensitivity to DDR1-selective inhibitors, suggesting a potential biomarker-guided therapeutic approach.

Boonto *et al*^[6]^ explored the regulation of metabolism in hepatocellular carcinoma by the long non-coding RNA *TUG1*. Their findings revealed that TUG1 promoted glycolysis by sponging miR-122-5p, thereby upregulating key glycolytic enzymes. This study highlights the pivotal role of lncRNAs in cancer metabolism and positions *TUG1* as a potential therapeutic target in hepatocellular carcinoma.

In a letter to the editor, Vats *et al*^[7]^ highlighted the prognostic significance of the chromosomal passenger complex (CPC)-cyclin/cyclin-dependent kinase (CDK) axis in lung adenocarcinoma. Their analyses revealed that elevated expression of CPC components was significantly correlated with shorter first progression survival, particularly in conjunction with S- and M-phase cyclin/CDK complexes. These findings suggest that the CPC-cyclin/CDK axis may serve as both a prognostic indicator and a potential therapeutic target in lung adenocarcinoma.

Kakurina *et al*^[8]^ conducted a pilot study to evaluate the clinical relevance of serum fascin-1 (FSCN1) in head and neck squamous cell carcinoma. They found that increased FSCN1 levels were associated with lymph node metastasis and disease progression within 12 months. Notably, FSCN1-positive circulating tumor cells were more abundant than those in primary tumors, supporting their role in metastasis. The study proposes serum FSCN1 as a non-invasive biomarker for predicting the progression and recurrence risk of head and neck squamous cell carcinoma.

Collectively, the studies in this issue illustrate the multifaceted nature of cancer biology and the importance of integrating molecular, clinical, and epidemiological approaches. From endocrine-cancer interactions to resistance mechanisms and metastatic progression, these contributions advance our knowledge and open new avenues for precision oncology.

We hope this special issue will inspire further research and foster translational efforts aimed at improving cancer diagnosis, treatment, and patient outcomes. We extend our gratitude to the authors, reviewers, and editorial team for their invaluable contributions to this issue.
